# The evolution of polarization in the legislative branch of government

**DOI:** 10.1098/rsif.2019.0010

**Published:** 2019-07-17

**Authors:** Xiaoyan Lu, Jianxi Gao, Boleslaw K. Szymanski

**Affiliations:** 1 Network Science and Technology Center and Department of Computer Science, Rensselaer Polytechnic Institute, Troy, NY 12180, USA; 2 Społeczna Akademia Nauk, Łódź, Poland

**Keywords:** dynamical system, political polarization, equilibrium, stability

## Abstract

The polarization of political opinions among members of the US legislative chambers measured by their voting records is greater today than it was 30 years ago. Previous research efforts to find causes of such increase have suggested diverse contributors, like growth of online media, echo chamber effects, media biases or disinformation propagation. Yet, we lack theoretic tools to understand, quantify and predict the emergence of high political polarization among voters and their legislators. Here, we analyse millions of roll-call votes cast in the US Congress over the past six decades. Our analysis reveals the critical change of polarization patterns that started at the end of 1980s. In earlier decades, polarization within each Congress tended to decrease with time. By contrast, in recent decades, the polarization has been likely to grow within each term. To shed light on the reasons for this change, we introduce here a formal model for competitive dynamics to quantify the evolution of polarization patterns in the legislative branch of the US government. Our model represents dynamics of polarization, enabling us to successfully predict the direction of polarization changes in 28 out of 30 US Congresses elected in the past six decades. From the evolution of polarization level as measured by the Rice index, our model extracts a hidden parameter–polarization utility which determines the convergence point of the polarization evolution. The increase in the polarization utility implied by the model strongly correlates with two current trends: growing polarization of voters and increasing influence of election campaign donors. Two largest peaks of the model’s polarization utility correlate with significant political or legislative changes happening at the same time.

## 1. Introduction

Conflict and consensus play important role in the functioning of a social system. In the context of political competition, they manifest themselves as complementary processes, one is polarization of opinions and the other is collaboration to reach consensus on shared national interests [1]. Polarization arises from the politicians’ need to represent opinions of their voters while collaboration is required to balance the interests of many groups. Numerous previous publications have focused on the role of social conformity [2–4] in polarization. Among these publications, many hypotheses have been proposed to explain the observed emergence of increased polarization, including social homophily [5], opinion dynamics [6], selective exposure [7], social bots [8], echo chambers [9,10], propagation of low-quality information or fake news [11,12], as well as the effect of viral news [13] and social media [14]. While these models study different aspects of polarization of political views, they share some common assumptions about human social behaviour [15], including the following: (i) individuals iteratively update their views to reach consensus with their neighbours in a social network; (ii) the tolerance of conflicting views is limited in social context, so frequent active disagreements usually break of social ties [16]. These assumptions indicate that the loyalty to one’s group usually leads to the conformity with views of the group’s majority [17], and such conformity tightens social ties within the group. Therefore, in our model, we allow the current polarization level to influence its future growth.

We analyse millions of roll-call votes cast in the US Congress [18] over the past six decades to identify the evolution of political polarization patterns. Using the roll-call vote results, we quantify the level of polarization in the legislative branch of government over the last six decades. We assume a social system dominated by two parties. In such a system, polarization and collaboration can convert into each other but they maintain their sum constant at 1. A simple model of the dynamics of competition between exclusive groups [19] can be extended to the case of non-exclusive competing groups whose members can convert polarization and collaboration into each other. The dynamics of the extended model can be written as

1.1 $$\frac{\text{d}x}{\text{d}t}=yP_{cp}(x,u_{\;p})-xP_{\;pc}(x,u_{\;p}),$$

where *x* ∈ [0, 1] is the current polarization level, as measured by the real legislative votes, while *u*_*p*_ is a parameter independent of the current polarization level. We call parameter *u*_*p*_ the polarization utility in analogy to the role of gravitational force in physics. The complementary values denoted as *y* = 1 − *x* and *u*_*c*_ = 1 − *u*_*p*_ represent the current collaboration level and the non-partisan utility, respectively, while *P*_*cp*_(*x*, *u*_*p*_) is the probability of collaboration converting to polarization per unit of time. For symmetry, *P*_*pc*_(*x*, *u*_*p*_) represents the probability of polarization converting to collaboration per unit of time. When two parties have exactly the same ‘Yes’ ratio on a bill, the collaboration level reaches its maximum *y* = 1. Studying polarization in the US Congress, we assume the evolution is fully governed by the nonlinear dynamics defined in equation (1.1). However, every 2 years, the dynamical system moves to a new state (determined by the election of members of the next Congress) that becomes the initial state for that Congress.

Up to the end of 1980s, the polarization level within each Congress tended to decrease. However, our results show that since then, the polarization has been likely to grow within two-year term of each Congress. This phenomenon is represented in our model by the change of the polarization utility, since it determines the polarization level to which the system converges, regardless of its initial state. The nonlinear dynamics of our model successfully predict the direction of polarization change in 28 out of 30 US Congresses for the past six decades. The two Congresses that are in disagreement with the model predictions have very small variations of polarization, with a weakly defined polarization direction, making the predication error small. Moreover, our results show that the further away is the initial polarization caused by member replacement from the corresponding equilibrium defined by our model, the higher is the speed with which the polarization level changes during the two-year period between elections.

In our model, the nonlinear gain–loss function quantifies the conversion between collaboration and polarization among legislators. The model implies that the polarization level always converges to an equilibrium point, while replacement of members in each Congress caused by election sets the new initial polarization level. We also derive an analytic expression for the equilibrium points, which are defined by the polarization utility and the system’s sensitivity to the current polarization. Our model implies that the observed increased polarization in the recent few decades is caused by the growing polarization utility. This conclusion prompts the question about the causes of this growth. We address this question in the Discussion section.

## 2. Quantifying polarization in the legislative branch

We analyse millions of roll-call votes cast in the US Congress [18] over the past six decades to identify evolution of political polarization patterns. The statistics of the roll-call votes in Congresses with sessions numbered from 85 to 114 are shown in table 1. This dataset contains approximately 7 million votes in both the Senate and the House of Representatives cast by a total of 1498 and 1395 legislators from Democratic and Republican Parties, respectively. We adopt the well-known Rice index [20] to measure party dissimilarity in legislative voting. The Rice index is defined as the mean absolute distance between the ‘Yes’ ratios of Democratic and Republican Parties on the *b*th bill

**Figure 1.**
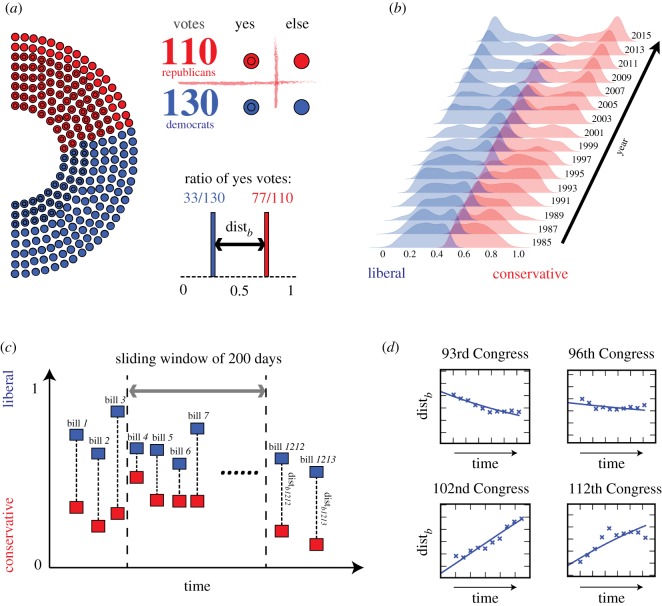
The illustration of the data collections and processing workflow. (*a*) For each bill voted in the Congress, we measure the mean absolute distance, dist_*b*_, between the votes of the Democratic and Republican Parties cast for the bill *b*; (*b*) the distribution of the political polarization measured using the roll-call votes within each Congress. Labels at the right corner of each sub-plot identify the first year of each Congress. In the 1980s and 1990s, the polarization levels are generally smaller than the levels in the 2000s and 2010s. After 2001, the two peaks of the voting results at the opposite ends of the political spectrum start to emerge, indicating the growth of the number of bills on which two parties strongly disagree; (*c*) regardless of the content of bills, we compute the average distance of bill votes between two parties within each Congress in a sliding window of 200 days (equation (2.2)); (*d*) the political polarization levels at 10 evenly distributed sampled time points exhibit an evolution of polarization patterns from one type of behaviour to another: the polarization level decreases in the 93rd Congress as the time from the replacement of members increases, while polarization remains at a relatively stable level in the 96th Congress, and grows in the Congresses with sessions numbered from 102 to 112. (Online version in colour.)

2.1 $${\text{dist}}_{b}=|E_{\left\{1\leq i\leq N_{\mathrm{Rep}}\right\}}{\text{Rep}}_{ib}-E_{\left\{1\leq j\leq N_{\mathrm{Dem}}\right\}}\;{\text{Dem}}_{\;jb}|,$$

where *N*_Rep_ and *N*_Dem_ denote the numbers of legislators from the Republican and Democratic Parties participating in the vote, while Rep_*ib*_ and Demo_*jb*_ are the votes cast by Republican *i* and Democrat *j* for bill *b*, respectively. E represents the corresponding average over all legislators in each party. The result of a vote is coded as 1 for ‘Yes’ and 0 otherwise because the bills pass by majority in both the Senate and the House of Representatives and therefore abstaining is effectively equivalent to opposing bill passage. This procedure is illustrated in figure 1*a*.

**Table 1.** The statistics of the roll-call votes from the 85th to the 114th US Congress. The bills which received less than 30 votes are not included in the above statistics.

party	#members	#bills	#votes	#votes/members	#votes/years
Democratic Party	1498	31 879	7 368 921	4919.17	122815.35
Republican Party	1395	31 879	6 275 886	4498.84	104598.1

Regardless of the content of bills, we compute the average distance of bill votes between the two parties over every 199 day intervals. Formally, the polarization level at the *t*th day of the *k*th Congress is quantified as

2.2 $$x_{k}(t)=E_{\left\{b:|t_{b}-t|<100\right\}}{\text{dist}}_{b},$$

where {*b* : |*t*_*b*_ − *t*| < 100} is the set of bills voted within 199 days centred at the *t*th day of the *i*th Congress and *i* starts at the 100th day of each Congress and ends 99 days before the last day of this Congress. Hence, each measurement includes exactly 200 days of voting. This step is illustrated by figure 1*c*. The averaging reduces the noise of the raw data because the topics of the legislative bills may differ day by day in each Congress, and there were several periods of times in the past six decades during which very few bills were voted on by the US Congresses. Compared to the approaches [21–23] defining the conflicting level between individuals, the Rice index defined in equation (2.1) reflects the general trend of behavioural partisanship while preserving its simplicity by quantifying polarization at party level. More importantly, the Rice index enables us to develop a dynamical equation, equation (1.1), which captures the macroscopic behaviour of the evolution of political polarization, regardless of the complex interactions between individual legislators considered in [21].

For every Congress and each bill, we measure the distance dist_*b*_ between two parties using equation (2.1). As dist_*b*_ ∈ [0, 1], the political preference for bill *b* is defined as *D*_*b*_ = 0.5 − dist_*b*_/2 for the Democratic Party and *R*_*b*_ = 0.5 + dist_*b*_/2 for the Republican Party. Then, using the kernel density estimation (KDE) [24], we evaluate the distribution of distances *D*_*b*_ and *R*_*b*_, of Democratic and Republican Parties, respectively, from the centre of the polarization range. Figure 1*b* shows the distribution of these distances which represent positions of the two parties regarding the bills voted in each Congress.

## 3. Dynamical model of political polarization

We assume a two-party political system, such as exemplified by the UK, but applicable also to the USA and other countries with a multi-party system dominated by two major parties. We also assume a social system in which polarization and collaboration can convert into each other. The polarization is reflected by the Rice index as defined in equation (2.2). And we define the complementary of polarization level as the collaboration level. A simple model of the dynamics of such conversion is given in equation (1.1) using the same notation as previously.

Following [19], we assume that *P*_*cp*_ has the following simple form:

3.1 $$P_{cp}(x,u_{\;p})=cx^{a}u_{\;p}$$

which is supported by the normative social influence theory [17] that postulates that the current polarization level *x* influences the probability of conversion. To preserve symmetry under the conversion from *y* to *x* and vice versa, we define *P*_*pc*_ as

3.2 $$P_{\;pc}(x,u_{\;p})=P_{cp}(y,u_{c})=cy^{a}u_{c}=c{\left(1-x\right)}^{a}(1-u_{\;p}).$$

Similar but not identical nonlinear gain–loss equations for the state dynamics have been successfully applied to model various types of polarization, ranging from religious affiliation [19], to language choice [25] and political affiliation [26]. The model introduced in [19] ‘idealizes a society as partitioned into two mutually exclusive social groups, X, the unaffiliated, and Y, those who adhere to a religion’. Furthermore, the model assumes the attractiveness of a group increases with the number of members and the perceived utility of the group, a quantity representing many factors including ‘the social, economic, political as well as security benefits derived from membership and the spiritual or moral consonance with a group’. In our case, the Congress members’ affiliation to different political ideologies may not be exclusive, like affiliation to a language or a religion is. Thus, we assume that in the legislative system polarization and collaboration may convert into each other. For each bill, the polarization is measured by the difference between the two parties’ ‘Yes’ ratios. Accordingly, we define the collaboration level *y* as complementary to polarization level *x*, setting, *y* = 1 − *x*. Another extension to the original model from [19] is the use of different *u*_*p*_ parameter for each individual Congress. This is motivated by the fact that the initial state for each Congress changes periodically, every 2 years when new members are elected. By contrast, the whole population affiliated with religions or languages can be assumed static, allowing the use of the same model parameters over time. In summary, the nonlinear dynamics defined here captures the conversion between polarization and collaboration during different periods which is not considered in [25]. The parameter *u*_*p*_ is independent of the polarization level *x* which varies as the time changes. This parameter reflects the internal perception of benefits that a legislator may gain by supporting polarizing opinion. Such support does not have to manifest itself immediately but rather in the future votes. The term *x*^*a*^ captures the effect of the current polarization level on the evolution. The speed of evolution is defined by the parameter *c* in our model.

**Figure 2.**
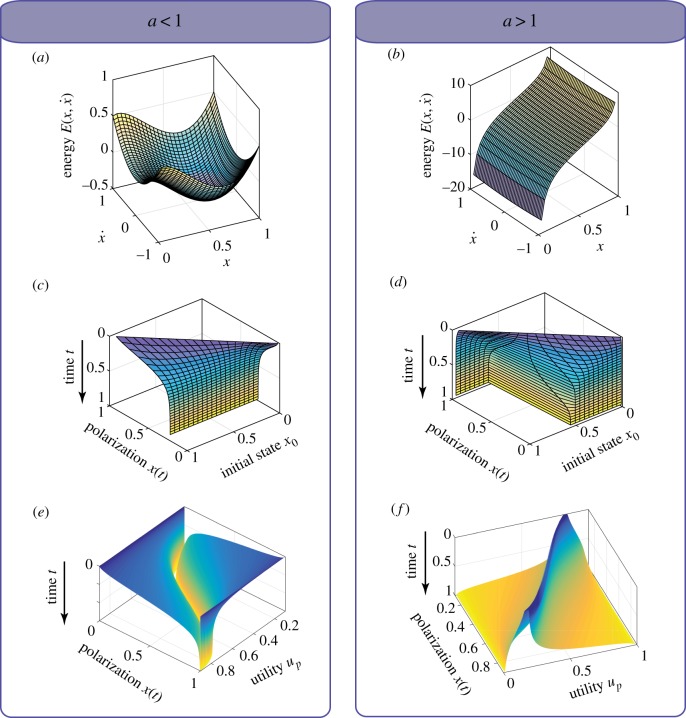
The convergence of the political polarization model in relation to the polarization utility *u*_*p*_ and initial state *x*_0_. The time *t* is normalized in the plots to the range from 0 to 1. (*a*) The total energy of the dynamical system in relation to the polarization *x* and its first-order derivative $\dot{x}$ for *a* < 1 (equation (3.3)); (*b*) the total energy of the dynamical system in relation to the polarization *x* and its first-order derivative $\dot{x}$ for *a* > 1 (equation (3.3)); (*c*) for *a* < 1 the dynamical system always converges to certain polarization level (*a* = 0.6); (*d*) for *a* > 1 the dynamical system reaches either complete consensus or complete polarization depending on the initial state *x*_0_ (*a* = 2.5). (*e*) When *a* < 1, the equilibrium points of the dynamical system are stable and the system gets trapped at these equilibrium points as the time approaches infinity; (*f*) when *a* > 1, the initial states (on the top of the hills) are the tipping points causing the system to converge either to 0 (full polarization) or 1 (full consensus) from its initial state *x*_0_. (Online version in colour.)

The total energy [27] in this dynamical system is governed by the following equation:

3.3 $$E(x,\dot{x})=\frac{1}{2}{\dot{x}}^{2}-cxy\left(\frac{u_{\;p}}{x^{1-a}}-\frac{u_{c}}{y^{1-a}}\right)$$

which is constant on the solution curves or trajectories of this system, i.e. $E(x,\dot{x})=C$ for some constant *C*. The total energy function $E(x,\dot{x})$ in relation to the current polarization *x* and its first-order derivative $\dot{x}$ is shown in figure 2*a* for *a* < 1 and figure 2*b* for *a* > 1.

Given the model parameters *a* and *u*_*p*_, besides the equilibrium points at x* = 0 and 1, the exact equilibrium points of the dynamical system defined by equation (1.1) can be derived as

3.4 $$x^{*}(u_{\;p},a)=\frac{1}{1+{\left(\left(1-u_{\;p}\right)/u_{\;p}\right)}^{1/\left(1-a\right)}}.$$

The derivation of the equilibrium points is provided in the electronic supplementary material.

The trajectories of the nonlinear dynamical evolution are shown in figure 2*c*–*f* where the *x*-axis represents time *t* and the *y*-axis represents the polarization. Each trajectory curve starts from its initial polarization level *x*_0_ which is represented by the *z*-axis. We show the surface of these convergence process for *a* < 1 and the initial polarization in full range from *x*_0_ = 0 to *x*_0_ = 1, in figure 2*e*. Likewise, figure 2*d* contains similar visualization results with *a* > 1 and initial polarization levels *x*_0_ at the tipping points, i.e. the points at the edges drawn in dark shading in figure 2*f* . The equilibrium points of the dynamical model are stable when *a* < 1. In such case, the dynamical system of equation (1.1) always converges to the x* ∈ [0, 1] after the sufficient period of evolution; figure 2*c*,*e* shows example with *a* = 0.6. By contrast, when *a* > 1, the final state of the dynamical system depends on the initial state and the position of the tipping point; figure 2*d*,*f* shows example with *a* = 2.5. The dynamical system will eventually converge to either *x* = 0 or *x* = 1.

In the context of political polarization, the case with *a* < 1 corresponds to a healthy political system which maintains the polarization level within a certain range. However, the case with *a* > 1, corresponds to a system which switches easily between fully polarized equilibrium point and complete consensus convergence point. In the US political system, a change of the system’s initial state is caused periodically every 2 years by the election of new legislators. The system near the tipping point at the end of one Congress is vulnerable to extreme state switching under even a small change of the initial polarization for the next Congress.

We simulate the nonlinear dynamics defined by equation (1.1) until the polarization level *x* converges. When *a* < 1, the equilibrium point is exactly the final polarization level of convergence. When *a* > 1, the only equilibrium points are *x* = 1, which corresponds to the full polarization, and *x* = 0, which represents the full consensus. Moreover, some of the initial states are unstable; they are the tipping points from which the dynamical system converges in a non-deterministic way to either of the two stable equilibrium points. We grid-search for the tipping points with different values of *u*_*p*_. In each iteration, we increase the value of *x* by a small Δ*x*. If the final convergence states have changed from one state to another due to the increment of Δ*x*, then the current *x* is identified as the tipping point.

## 4. Evolution of political polarization patterns

We fit the political polarization model defined by equation (1.1) to the data points *x*_*i*_(*t*) which represent 10 evenly distributed in time sampled polarization levels at *t*_*i*_ = *t*_*i*,1_, *t*_*i*,2_, …, *t*_*i*,10_ of the *i*th Congress. The system is assumed to have the universal *a* and *c* values at all times, but each Congress *i* has its own parameter *u*_*p*_ defined as the polarization utility, and the particular initial state, *x*_*i*,0_, which is defined by the member replacements caused by the most recent election. In other words, the nonlinear dynamics here only model the evolution of polarization during a constant period of time between elections. Member replacements in each Congress resulting from election move the dynamical system to a new state which is the new Congress’s initial state. As seen in equation (1.1), the polarization utility *u*_*p*_ does not change over time like the current polarization level *x* does. We assume that each Congress has a consistent polarization utility as long as the same members stay. Therefore, the polarization utility is set as a fixed value for each Congress, independent of the evolving polarization level within each two-year term.

For the *i*th Congress, given parameters *a* and *c*, we grid-search the possible *u*_*i*,*p*_ and *x*_*i*,0_ values to minimize the least absolute errors (LAE) between the real polarization level [*x*_*i*_(*t*_*i*,1_), *x*_*i*_(*t*_*i*,2_), …, *x*_*i*_(*t*_*i*,10_)] and theoretical polarization level found by numerical simulation of equation (1.1) at the corresponding time points. Next, we find the optimal values of *a*, *c* values which minimize the sum of the LAEs of all Congresses. Then, we repeat the grid-search for local and global parameters by narrowing neighbourhood of parameter values so each repetition increases the precision of the results. We stop when precision reaches 0.01.

**Figure 3.**
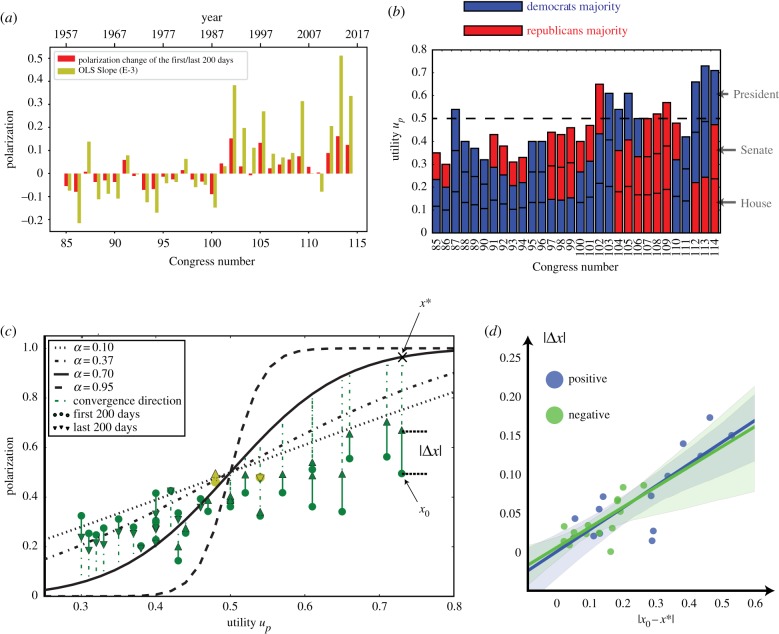
The evolution of the political polarization in the US Congress with subsequent sessions numbered from 85 to 114. (*a*) Change of polarization within each US Congress based on data and the optimal slope parameters estimated by the Ordinary Least Squares (OLS) linear regression given the 10 evenly distributed in time points of polarization sampling for each Congress. The polarization level is likely to decrease within each Congress after the member replacement in the 1970s and 1980s. However, after the 101th Congress, which started in 1989, the polarization level is likely to increase instead; (*b*) the estimated values of the polarization utility *u*_*p*_ are generally increasing, while the periods of sharp growth are often associated with the change of majorities in the Senate and the House of Representatives; (*c*) when the initial polarization level (dark circles) is smaller than the stable polarization level predicted by our model (solid black curve), we observe an increase of polarization within the corresponding Congress. The direction of such change in 28 out of all 30 Congresses are explained by the model (dark arrows), while the two Congresses in disagreement with prediction have minimal variations of the polarization level (light arrow markers) which indicates weakly formed polarization direction; (*d*) when the initial polarization level *x*_0_ of a Congress is farther away from the corresponding equilibrium point, the absolute change of polarization during the two-year term, i.e. |Δ*x*|, of this Congress is usually higher than of Congresses in which the initial polarization levels are closer to the equilibrium points. The colour of the scatter points indicates the sign of Δ*x*. (Online version in colour.)

The inference procedure estimates the values of universal parameters at *a* = 0.7, *c* = 0.37 while the set of values for *u*_*p*_, *x*_0_ is shown in figure 3*b*,*c*. In figure 3*b*, we illustrate the estimated value *u*_*i*,*p*_ of the *i*th Congress. In general, this value increases as the Congress session numbers grow from 85 to 114. The black solid curve in figure 3*c* shows the equilibrium points for different *u*_*p*_ values with global parameter *a* estimated from the voting data. To illustrate the impact of parameter *a* on values of the equilibrium points, the dotted lines represent these values for *a* = 0.1, *a* = 0.37 and *a* = 0.95, respectively. This figure also illustrates that most of the Congresses last too short for the system to reach the equilibrium point before the election of new members redefines the new starting point of the system for the next Congress.

Since the estimate *a* = 0.7 is smaller than 1, the dynamical system has a unique equilibrium point for any given *u*_*p*_. As shown in figure 3*c*, when the polarization level *x* is larger than the stable equilibrium point x*, this level decreases to approach this point. This explains why the polarization level decreases in most of the Congresses in the 1970s and 1980s. After the member replacement caused by the election, the initial polarization levels *x*_0_’s during this period were usually higher than the corresponding stable equilibrium points of the dynamical system (figure 3*c*). Therefore, the polarization level decreases within the two-year term Congress. By contrast, when the equilibrium point x* is larger than the initial polarization *x*_0_, the polarization *x* increases over time to approach the equilibrium point. This explains why the polarization level increases in most Congresses after the 1980s. Hence, the observed polarization patterns are fully recreated by the dynamics of our model.

It is worth noting that the initial polarization levels of the Congresses in the 1990s are actually not significantly higher than those observed in the previous Congresses. However, the polarization utility *u*_*p*_ has become larger and in the later decades exceeded 0.5 as figure 3*b* and table 2 indicate. Consequently, the polarization levels at the end of Congresses have significantly increased. This explains the rapid growth of polarization in the later Congresses. The sudden growth of polarization utility in the 101st and 102nd Congresses (1989–1993), revealed by our model is in agreement with [21] which describes a dramatic change of polarization that started during Clinton’s term (1993–1994), and solidified during the 104th Congress (1995–1996). Thus, the growth of polarization utility came right before the growth of polarization because legislators need time to adjust voting to the increased polarization utility. As seen in figure 3*d*, the absolute change of polarization |Δ*x*| grows as the distance between the initial state *x*_0_ and equilibrium point x* increases. If the initial polarization level of a Congress is far away from its final point of convergence, then the rapid change of polarization would be expected within the two-year term. The direction of such change in 28 out of all 30 Congresses is explained by the model, while the two Congresses in disagreement with the model prediction have very small variations of polarization level as shown by the light markers in figure 3*c*.

**Table 2.** The estimated values of the polarization utility *u*_*p*_ in each Congress. The 14 Congresses with the presidential election held in the preceding year increase *u*_*p*_ on average by 11.3% compared to the corresponding previous Congresses while the 15 Congresses with midterm elections (during which the President passes half of his term), decrease the polarization utility *u*_*p*_ by −1.5% on average. Moreover, in the first three decades, the average polarization utility grew slowly by 0.056, so 18.1% on average in Presidential election Congresses. All this growth was gained in four Presidential elections in which the newly elected President and his predecessor belonged to different parties; each of these elections contributed growth of 0.115 so 36.4%, on average. By contrast, the polarization utility decreased by −0.043 or −9.3% in midterm election Congresses, raising only 14.3% over 30 years. In the latest three decades, the polarization utility grew in both types of Congresses with similar average rates, of 0.023 or 4.6% for midterm election Congresses, and 0.21 or 7.3% for Presidential election Congresses. From the 100th Congress to 114th Congress the polarization grew 77.5%, so five times higher than in the earlier period. Notably, 6 of 14 Presidential election Congresses are associated with the polarization utility *u*_*p*_ > 0.5 while only one of 15 midterm election congresses had such high polarization utility.

midterm election congresses				presidential election congresses			
number	*u*_*p*_	change	percentage (%)	number	*u*_*p*_	change	percentage (%)
86th	0.3	−0.05	−14.3	*87*th*	*0.54*	0.24	80.0
88th	0.4	−0.14	−25.9	89th	0.37	−0.03	−7.5
90th	0.32	−0.05	−13.5	91st	0.43	0.11	34.4
92nd	0.38	−0.05	−11.6	93rd	0.31	−0.07	−18.4
94th	0.33	0.02	6.5	95th	0.4	0.07	21.2
95th	0.4	0.00	0.00	97th	0.44	0.04	10.0
98th	0.43	−0.01	−2.3	99th	0.46	0.03	7.0
100th	0.4	−0.06	−13.0	101st	0.47	0.07	17.5
102nd	0.65	0.18	38.3	103rd*	*0.61*	−0.04	−6.2
104th	0.54	−0.07	−11.5	105th*	*0.61*	0.07	13.0
106th	0.5	−0.11	−18.0	107th*	*0.5*	0.00	0.0
108th	0.52	0.02	4.0	109th*	*0.57*	0.05	9.6
110th	0.48	−0.09	−15.8	111th	0.42	−0.06	−12.5
112th*	*0.66*	0.24	57.1	113th*	*0.73*	0.07	10.6
114th	0.71	−0.02	−2.74				
86th–100th	0.370	−0.043	−9.3		0.421	0.056	18.1
101st–114th	0.580	0.021	7.3		0.559	0.023	4.6
All	0.468	−0.013	−1.5		0.481	0.039	11.3
	4 positive, 10 negative, 1^a^				9 positive, 4 negative, 6^a^		

^a^Denotes the polarization utility *u*_*p*_ over 0.5, marked by italic font in the *u*_*p*_ column.

Another sign of changed polarization patterns is the number of Congresses in which initial polarization utility is at least 50%, making it equal to or stronger than the collaboration utility as defined in equation (1.1). Only one Congress among 15 in the first three decades reached this level, while it was achieved by 11 out of 14 Congresses in the last three decades.

In summary, our model explains the observed polarization patterns. In the 1970s and 1980s, the initial polarization *x*_0_ is generally larger than the stable polarization x*, so we observe a decrease of polarization within each of two-year term Congress. In other words, the legislators gradually agree more and more with the members of the opposite party than initially, while they held more conflicting views at the very beginning of each Congress session. After the 101st Congress (1989–1991), the initial polarization *x*_0_ is generally smaller than the stable polarization level x*. Therefore, during the corresponding Congresses, the polarization *x* increases to approach the corresponding equilibrium point defined by the given *u*_*p*_. This trend matches the transition observed during the 103rd and 104th Congresses [21], when the moderate members of each party joined their majority-party coalitions, leaving the middle ground deserted.

## 5. Discussion

As discussed in [28], in the past the polarization in the Congress was higher in the early part of the Congress term during which procedural issues were voted and it is lower in the later part. This past pattern led to a hypothesis that ‘Lion’s share of party polarization in Congress can be accounted for by the changing dynamics of voting on procedures’ [28]. The first contribution of our paper is the analysis of voting that uses the Rice index to show this hypothesis does not hold anymore. Over the last 14 Congresses this pattern reversed, and polarization increases in the late part of each Congress. Our second contribution is to extend the model from [25] to non-exclusive and dynamically changing competing groups. The third is to recover the model parameters from the roll-call vote data to minimize the difference between the model and the data.

The polarization utility introduced here and its change over time explain observed polarization of each Congress in the last 60 years with high accuracy. Hence our dynamical model sheds some light on the causes of increased polarization in recent decades. As seen in figure 3*c*, when *u*_*p*_ is small, the resulting stable polarization level decreases as the value of *a* increases, in response to the decreased probability of conversion from collaboration to polarization (cf. equation (1.1)). In such case, the dynamical system is less sensitive to the current polarization level *x* because the term *x*^*a*^ in equation (1.1) becomes smaller as *a* grows. Therefore, when *u*_*p*_ is small, a large value of *a* decreases the polarization level. However, when the value of *a* exceeds 1, some initial states become tipping points, from which the system evolves in a non-deterministic way towards one of the two possible extreme equilibrium points, one of which, *x* = 1, corresponds to the full polarization, while the other, *x* = 0, represents the full consensus. Below the tipping point, the system converges to one of these two points, and above, it evolves towards the other. In the US political system, a change of the initial system state happens periodically every 2 years when the new legislators are elected. The system in the neighbourhood of a tipping point is vulnerable to even small change in initial polarization that may switch the system convergence point from one extreme equilibrium point to another. Such abrupt and radical equilibrium point switching is absent when *a* is smaller than 1.

According to our model, we witness a growth of the polarization utility, *u*_*p*_, over the recent decades. The question arises what are the causes of this increase. To answer this question, we start by observing that a politician needs to be elected to become legislators and repeatedly reelected to continue in this role. Thus, the polarization utility for them lies in its ability to bring votes. This can be accomplished either directly, by representing voters’ opinions, or by gaining resources for election campaign funding. In the case of direct support, the polarization of voters has been rising in recent decades for such reasons as the echo chambers effect [9,10] and the growth of new, often strongly biased social media [14] or spread of misinformation [11,12]. Voters increasing polarization raises the polarization utility, which is indirectly reflected as the phenomenon that legislators align their voting with positions of their electorates. The indirect support is gaining in importance because of escalating costs of political campaigns fuelled by the growing numbers of effective advertising channels and raising costs of advertising [29].

To corroborate this conclusion, we identified two largest jumps in polarization utility resulting from election of Congress members (table 2). The first jump of 0.24 happened in 1960 so it coincides with the start of civil right movement, increasing the US involvement in the Vietnam War, and generational changes in politics. In [22], the author observe that such ‘takeoff situations’ significantly increase polarization in the network structures of political connections. The second jump happened in year 2010, when the Supreme Court approved Super PACs which are allowed to collect unlimited contributions from many sources and to advocate for or against political candidates. Taming the causes of increased polarization is difficult. For example, requiring biased social media to provide time to advocates of the opposing opinions was shown to be counter productive [30].

Several interesting questions arise in relation to the presented work, that chart the paths for future research. The three of those are briefly discussed below. The first question arises about how much, if at all, polarization trends differ between the House of Representatives and the Senate. Our preliminary checking reveals that fitting the model parameters to the House votes yields *c* = 0.38 and *a* = 0.6, which is close to the results for both Senate and House together, *c* = 0.37 and *a* = 0.7. However, terms of representative and senators differ so in the future work we plan to study this issue. The second question is about differences in polarization dynamics between the Republican and Democratic Parties. To answer this question requires analysis of the bills that are voted on to measure how consistent each party is in its voting patterns. Again, we defer researching this interesting question to future work. Finally, the third question is related to the implications of the model introduced here that high polarization of voters makes the polarization utility higher to legislators. This trend may lead to higher polarization in the legislative chambers of government. The question arises under what conditions the polarized politicians may in turn influence their electorates to become polarized even more. Finding these conditions is important because should such feedback loop arise, it might destabilize democracy. In our future research, we will attempt to address this question by developing a quantitative model of polarization dynamic of voters that to larger extent than for legislators is shaped by the economic factors [31] and public opinions [32].

## Supplementary Material

Supporting Material The evolution of polarization in the legislative branch of government

## Data accessibility

All data needed to evaluate the conclusions in the paper are available from the website listed in [18].
