# Supplementary material for: The evolution of polarization in the legislative branch of government
Source: J R Soc Interface. 2019 Jul 17;16(156):20190010. doi: 10.1098/rsif.2019.0010 (PMC6685022; doi:10.1098/rsif.2019.0010)
Supplement: Supporting Material The evolution of polarization in the legislative branch of government [file rsif20190010supp1.pdf]

Supporting Material
The evolution of polarization in the legislative branch of government

Xiaoyan Lu[1], Jianxi Gao[1] and Boleslaw K. Szymanski[1,2]

[1]Network Science and Technology Center and Department of Computer Science, Rensselaer Polytechnic Institute, Troy NY 12180, USA
[2]Społeczna Akademia Nauk, Łódź, Poland

Derivation of the equilibrium points

The equilibrium points x^* of the dynamical model defined by Eq. [1.1] satisfy the following equation:

$$(1-x)x^a u_p = x(1-x)^a(1-u_p).$$

By putting all elements with x on LHS and u_p on RHS and taking into account that $x^{a-1} = 1/x^{1-a}$, we get

$$\left(\frac{1-x}{x}\right)^{1-a} = \frac{1-u_p}{u_p}$$

Then, taking both sides to power $1/(1-a)$ we get

$$\frac{1}{x} - 1 = \left(\frac{1-u_p}{u_p}\right)^{\frac{1}{1-a}}$$

Finally, moving -1 to RHS and inverting both sides we get the solution

$$x = \frac{1}{1 + \left(\frac{1-u_p}{u_p}\right)^{\frac{1}{1-a}}}$$

When $a < 1$, these equilibrium points are stable [1]. However, when $a > 1$, there are some initial states that are tipping points from which the system non-deterministically converges to one of the two extreme equilibrium points, either to full consensus or to full polarization. In the context of political polarization, the existence of tipping points changes the stability of the polarization evolution. When $a > 1$, and with the system in the neighborhood of a tipping point, in response to even a small perturbation of the system state, the system may change the convergence from one extreme equilibrium point to another. When $a < 1$, the dynamical social system is more robust against the random perturbations because it always converges to the equilibrium point with some level of polarization.

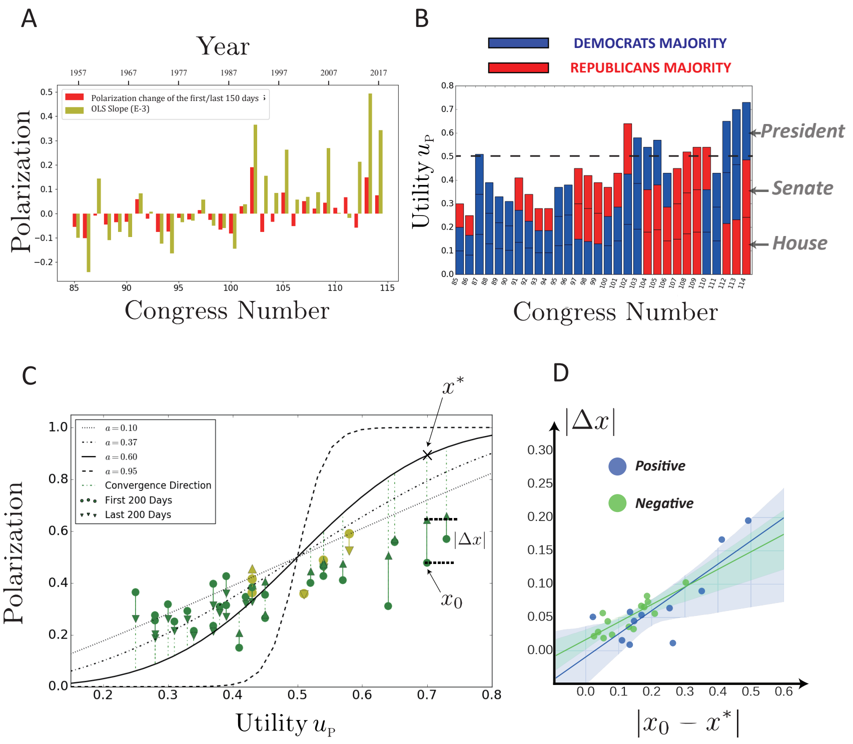

Figure 1: The evolution of the political polarization in the U.S. Congress with subsequent sessions numbered from 85 to 114. Results are re-produced using a sliding window of 150 days.

Impact of sliding window length

In our experiments, we extract the average polarization level by Eq. [2.2] using a sliding window of 200 days. Hence, each measurement includes exactly 200 days of voting. We test the sensitivity of the results to the choice of the sliding window length.

For a sliding window of 150 days, we re-produce Fig. 1 mirroring the Fig. 3 in the main content. Fig. 1(A) shows that the change of the polarization evolution also occurs at the 101th Congress. The estimated values of the polarization utility u_p are also generally increasing as seen in Fig. 1(B) while the periods of sharp growth are often associated with the change of majorities in the Senate and the House of Representatives. In Fig. 1(C), the direction of polarization change in 25 out of all 30 Congresses are explained by the model (green arrows), while only five Congresses are in disagreement with prediction. However, their variations of polarization levels (yellow arrow markers) are minimal, indicating weakly formed polarization direction. In Fig. 1(D), both the distance from the initial polarization level x_0 to the corresponding equilibrium point and the absolute change of polarization during the two-year term, i.e. $|\Delta x|$, for this Congress exhibit the similar correlation.

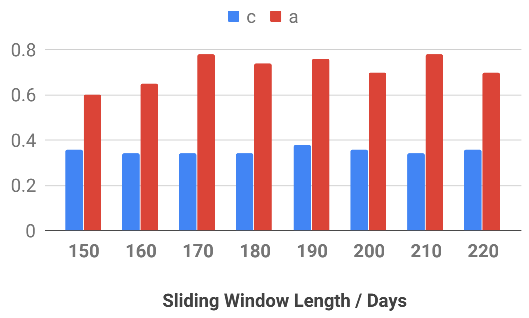

Figure 2: The LAE estimates of the model parameters a and c using different sliding window lengths.

Using different sliding window lengths, we re-estimate the model parameter by minimizing the LAEs. As illustrated in Fig. 2, we obtain the estimates which are all close to the results for a sliding window of 200 days ($c = 0.37, a = 0.7$).

References

[1] Abrams D, Yaple H, Wiener R. Dynamics of social group polarization: modeling the decline of religious affiliation. *Physical Review Letters*. 2011;107(8):088701.
